# Determinants of pain and activity limitations in foot osteoarthritis: An exploratory cross-sectional study in the Amsterdam-foot cohort

**DOI:** 10.1016/j.ocarto.2020.100134

**Published:** 2021-01-06

**Authors:** V.F.M. Ryman, M. van der Esch, J. Dekker, L.D. Roorda, J. van Dieën, J.W.R. Twisk, S.K. Verberne, E. Huijbrechts, W.F. Lems, M. van der Leeden

**Affiliations:** a Amsterdam Rehabilitation Research Centre, Reade, Amsterdam, the Netherlands; b Centre of Expertise Urban Vitality, Amsterdam University of Applied Sciences, Amsterdam, the Netherlands; c Amsterdam UMC, Vrije Universiteit, Department of Rehabilitation Medicine, Amsterdam UMC, Amsterdam, the Netherlands; d Vrije Universiteit Amsterdam, Department of Human Movement Sciences, Amsterdam Movement Sciences, Amsterdam, the Netherlands; e Amsterdam UMC, Vrije Universiteit, Department of Epidemiology and Biostatistics, Amsterdam Public Health Research Institute, Amsterdam, the Netherlands; f Fontys University of Applied Sciences, Department of Allied Health Professions, Eindhoven, the Netherlands; g Amsterdam Rheumatology and Immunology Center, Reade, Amsterdam, the Netherlands; h Amsterdam UMC, Vrije Universiteit, Department of Rheumatology, Amsterdam Movement Sciences, Amsterdam, Amsterdam, the Netherlands

**Keywords:** Osteoarthritis, Foot, Ankle, Pain, Activity limitations

## Abstract

**Objectives:**

Osteoarthritis (OA) of the foot-ankle complex is understudied. Understanding determinants of pain and activity limitations is necessary to improve management of foot OA. The aim of the present study was to investigate demographic, foot-specific and comorbidity-related factors associated with pain and activity limitations in patients with foot OA.

**Methods:**

This exploratory cross-sectional study included 75 patients with OA of the foot and/or ankle joints. Demographic and clinical data were collected with questionnaires and by clinical examination. The outcome variables of pain and activity limitations were measured using the Foot Function Index (FFI). Potential determinants were categorized into demographic factors (e.g., age, sex), foot-specific factors (e.g., plantar pressure and gait parameters), and comorbidity-related factors (e.g., type and amount of comorbid diseases). Multivariable regression analyses with backward selection (p-out≥0.05) were performed in two steps, leading to a final model.

**Results:**

Of all potential determinants, nine factors were selected in the first step. Five of these factors were retained in the second step (final model): female sex, pain located in the hindfoot, higher body mass index (BMI), neurological comorbidity, and Hospital Anxiety and Depression Scale (HADS) score were positively associated with the FFI score. The explained variance (*R*^*2*^) for the final model was 0.580 (adjusted *R*^*2*^ ​= ​0.549).

**Conclusion:**

Female sex, pain located in the hindfoot, higher BMI, neurological comorbidity and greater psychological distress were independently associated with a higher level of foot-related pain and activity limitations. By addressing these factors in the management of foot OA, pain and activity limitations may be reduced.

## 1 Introduction

Osteoarthritis (OA) is a common, degenerative, and debilitating disease. Despite its high prevalence and the individual and societal burden of the disease, OA of the foot has not been studied extensively, in contrast to OA of the knee and hip [[1], [2], [3], [4]]. OA of the foot can occur at many sites; however, the most common sites are the first metatarsophalangeal joint, the midfoot joints, and the ankle joint [1,2,5]. Since OA cannot be cured, current treatment options are aimed at reducing foot pain and activity limitations.

Adequate treatment of foot pain and activity limitations in patients with foot OA requires knowledge of its determinants. Although the number of existing studies on determinants is low, several factors appear to be related. Some studies have shown that demographic factors such as older age [6], female sex, and lower educational level in patients with foot OA [7] are associated with pain and activity limitations. These factors have also been shown to be associated with worsening of pain and activity limitations in knee and hip OA [8,9]. In addition to these factors, foot-specific factors, such as the location and pattern of foot symptoms, radiographic joint damage, and plantar pressure distribution may also affect pain and activity limitations. However, the evidence for these foot-specific factors is inconclusive or conflicting [[10], [11], [12], [13], [14]]. Finally, factors related to comorbidity, such as a high body mass index (BMI), psychological distress, diabetes, concomitant pain in other weight-bearing joints, and a higher comorbidity count have been associated with pain and activity limitations in foot and/or ankle OA [5,7,15]. Associations between comorbidity and clinical outcomes have also been found in knee and hip OA [8,9,16,17].

The limited number of studies does not provide a clear picture of the factors related to pain and activity limitations in this patient population. Therefore, the aim of this study was to investigate the associations of demographic, foot-specific and comorbidity-related factors with pain and activity limitations in patients with foot OA.

## 2 Method

A cross-sectional study using data from the Amsterdam-foot (AMS-foot) cohort was conducted.

### 2.1 Participants

The cohort consisted of patients, 18 years or older, who had been referred to a physician or podiatrist in the foot care clinic of an outpatient rehabilitation center (Reade, center for Rehabilitation and Rheumatology, Amsterdam, The Netherlands). Exclusion from the cohort occurred in cases where language barriers did not allow patients to complete the questionnaires. Data collection was performed by a trained research assistant after the first visit to the rehabilitation physician, and prior to the first visit to the multidisciplinary foot care clinic [18] except for data from medical records, which were gathered at a later stage.

Patients included in the present study had OA, based on a combination of radiographic evidence and pain, in at least one foot and/or ankle joint, had questionnaire data available, and provided informed consent. Patients with rheumatic comorbidities of the foot affecting pain and/or activity limitations (e.g., rheumatoid arthritis) were excluded from the analyses. Data collected between 2011 and 2019 were analyzed. Ethical approval was obtained from the medical ethical committee of the Slotervaart Hospital/Reade Amsterdam (registered under P1441). All data were kept confidential and the study was conducted in accordance with the Declaration of Helsinki [19].

## 3 Materials and procedure

### 3.1 Outcome variable

Pain and activity limitations were measured using the Foot Function Index (FFI) [20]. The FFI is a 23-item questionnaire, assessing three different areas: pain (9 items), disability (9 items) and activity restriction (5 items). For the present study, the modified FFI was used, where each item is scored on a 5-point Likert scale (0 meaning no pain/no difficulty/never and 4 meaning intense pain/impossible/always), with an additional option of “not applicable” [21]. The score was recalculated to a range from 0 to 100, where a higher score indicates more severe pain and activity limitations. For the activity restriction subscale, many “not applicable” responses were selected. Therefore, this subscale was not included in the recalculation, leaving the items of the pain and activity limitations subscales. We found that these subscales were highly correlated (Pearson’s correlation 0.752), and therefore the total score was used in the analyses.

### 3.2 Potential determinants

Potential determinants were selected based on the literature available, as well as the opinions of clinical experts. Determinants were divided into three subcategories: demographic factors, foot-specific factors, and comorbidity-related factors.

### 3.3 Demographic factors

Demographic factors included age (years), sex, educational level (no/primary education, secondary education, or higher education) and marital status (single or not single). These data were collected using self-administered questionnaires.

### 3.4 Foot-specific factors

Foot-specific factors included pain located in the forefoot, midfoot or hindfoot, foot deformities, location of radiographic OA (ROA) in the foot, and plantar pressure.

Pain location: Data on pain located in the forefoot, midfoot, and/or hindfoot were collected by the research assistant. It was reported as the presence or absence of current pain in the toes and/or forefoot (category forefoot pain), midfoot (category midfoot pain), and hindfoot and/or ankle (category hindfoot pain) and was marked on a standardized question grid by the research assistant.

Foot deformities: Foot deformities were recorded by the research assistant using the Platto-score. The Platto-score is used to quantify forefoot deformity (range 0–12) and hindfoot deformity (range 0–7) [22]. For the purpose of this study, the total score for the entire foot was used, and was standardized to range from 0 to 100, with a higher score indicating more deformities.

Location of ROA: Data on location of ROA in the foot were collected through assessment of x-ray reports in the patients’ medical records and recorded as present or not present in the forefoot, midfoot, and/or hindfoot. The x-ray assessments were made by radiologists.

Plantar pressure: Plantar pressure data were collected using an EMED-nt pedograph platform (Novel Electronics, Novel gmbh, Munich, Germany) (4 sensors per cm2, sampling frequency 50Hz), mounted in a 3.6 ​m walkway. A two-step protocol has previously shown good reproducibility [23], and was therefore used. Patients stood two steps away from the platform, contacting the platform on the second step. After the familiarization rounds, the measurements started and were repeated until three valid measurements (with the entire foot on the platform in a normal step, determined by research assistant and patient) had been recorded. The data were immediately analyzed using the EMED system software (Novel Ortho, Novel-Win). A division mask identified the different regions of the foot, which were subsequently divided into forefoot, midfoot, and hindfoot. Peak pressure (PP) and pressure time integral (PTI) were recorded for the entire foot as well as separately for the forefoot, midfoot and hindfoot. Contact time (CT) for the entire foot was also recorded. The values from the most affected foot (as reported by the patient) were used in the analyses. In the event both feet were equally affected, the average value of the two feet was used. PP was defined as the highest pressure value measured at each region of interest, expressed in kilopascal (kPa). PTI was defined as the integral of PP over time measured by the same sensors as the PP, expressed in kilopascal multiplied by seconds (kPa∗s).

### 3.5 Comorbidity-related factors

Comorbidity related factors included BMI, comorbid pain in the knees or hips, comorbidity count, specific comorbidity groups, and symptoms of anxiety and/or depression.

BMI: BMI was calculated from recordings of the patient’s height (m) and weight (kg).

Comorbid pain in lower extremities: Data on comorbid pain in the knees and/or hips were recorded using a self-administered questionnaire.

Comorbidity count: The comorbidity count was based on a self-administered questionnaire, adapted from the Health Interview Survey of Statistics Netherlands [24]. The Health Interview Survey covers twelve groups of chronic conditions, that are relatively the most prevalent in the Netherlands. The patients reported whether they had any of the following comorbidities: previous heart attack, another heart condition, atherosclerosis, hypertension, another circulatory condition, chronic obstructive pulmonary disease, another respiratory condition, serious conditions of the colon, another bowel condition, incontinence, another condition of the kidneys, bladder or urinary tracts, psoriasis, eczema, cerebrovascular accident, loss of sensation in the feet, another sensation defect of the feet, migraine, vertigo, diabetes, cancer, edema, or any other condition. The number of present conditions per patient was summed, making up the comorbidity count.

Specific comorbidity groups: Specific comorbidity groups were based on the disease areas of the Cumulative Illness Rating Scale questionnaire [25] (albeit with a dichotomous yes/no for each comorbidity group instead of a severity rating). The comorbidity categories were cardiorespiratory, gastrointestinal, urogenital, dermatologic, neurologic, and other.

Anxiety or depression: Symptoms of anxiety and/or depression were assessed using the Hospital Anxiety and Depression Scale (HADS). HADS is a self-administered questionnaire with fourteen items to be answered on a five-point scale (0–4); seven items on the anxiety subscale and seven items on the depression subscale, giving a score ranging from 0 to 42 (0–21 per subscale) with a higher score indicating more symptoms/signs of anxiety and/or depression [26]. The total score was used in the analyses.

### 3.6 Statistical analyses

Statistical analyses were carried out using IBM SPSS Statistics version 24. Normality was checked for all continuous variables by visual inspection of histograms. Descriptive data were then reported using mean (SD) (normally distributed) or median (IQR) (not normally distributed) for continuous variables, and frequency (N [%]) for categorical variables.

The analyses were performed in two steps. In step one, multivariable regression analyses were performed for each sub-category (i.e., demographic, foot-specific, and comorbidity-related) using a stepwise backward selection method, excluding variables with a p-value ≥0.05. The dependent variable was the FFI total score. The independent (predictive) variables were the factors in each sub-category (i.e., demographic, foot-specific and comorbidity-related). In the second step, multivariable linear regression analyses with backward selection were performed using all factors from step one with a p-value <0.05. Factors with a p-value < 0.05 were retained in the final model. Finally, a bootstrapped multivariable linear regression analysis using 2000 samples was performed on the final model. The results were reported as unstandardized regression coefficients (B) and 95% bias-corrected and accelerated confidence intervals (95%BCaCI), as well as p-values. Additionally, the R^2^ and adjusted R^2^ were reported.

## 4 Results

**Fig. 1:**
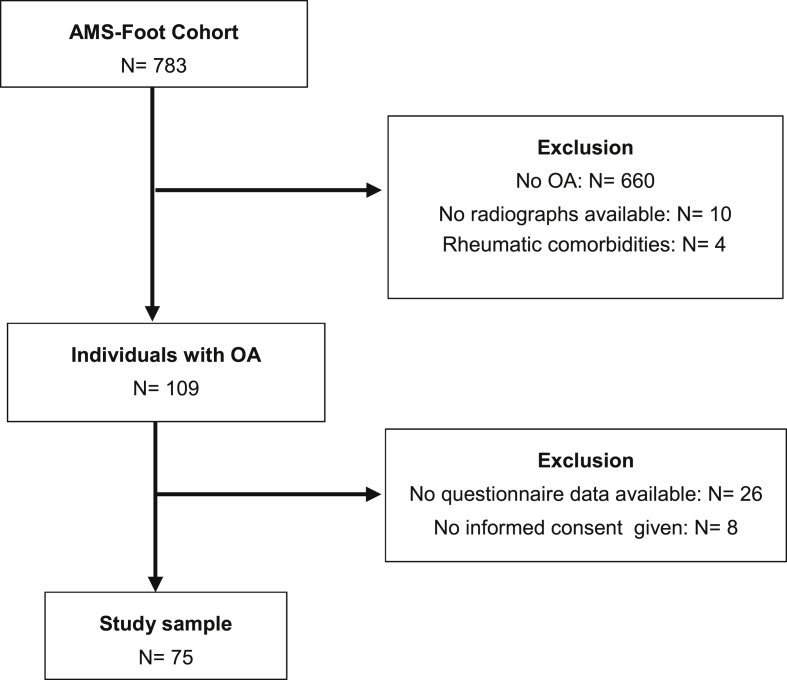
Flow chart of selection process.

A total of 75 patients were included in the study (see Fig. 1 for a flow chart of the selection process). Table 1 provides information on patient characteristics. Although the majority of the plantar pressure variables were not normally distributed, mean values are presented to allow for comparison with data from other studies. All PP variables were highly correlated with the corresponding PTI variables (Pearson correlations between 0.72 and 0.86), as shown in previous studies [27,28]. Therefore, all PTI variables were excluded from the analyses. Missing values in the data were excluded through pairwise deletion.

**Table 1:** Patient characteristics.

Variable	Mean ​± ​SD	Range	*n*
Outcome variable			
**FFI total**	43.9 ​± ​19.7	10.7–83.3	74
**FFI pain subscale**	47.4 ​± ​20.6	8.3–88.9	72
**FFI disability subscale**	45.7 ​± ​23.1	0–88.9	74
Demographic factors			
**Age (yrs)**	64.4 ​± ​9.5	41–82	75
**Female (%)**	80.0		75
**Marital status, single (%)**	34.7		75
**Educational attainment (%)**			75
**No/Primary education**	8.0		
**Secondary education**	57.3		
**Higher education**	34.7		
Foot-specific factors			
**Pain locations in the foot (%)**			75
**Forefoot**	86.7		
**Midfoot**	74.7		
**Hindfoot**	64.0		
**Plantar pressure**			
**PP, kPa**			67
**Total**	699.7 ​± ​332.0	33.4–1275.0	
**Forefoot**	779.9 ​± ​277.7	216.7–1275.0	
**Midfoot**	179.6 ​± ​100.4	40.0–571.7	
**Hindfoot**	423.9 ​± ​237.8	161.7–1275.0	
**PTI, kPa∗s**			67
**Total**	363.5 ​± ​208.8	12.9–1085.7	
**Forefoot**	316.1 ​± ​160.5	113.6–930.5	
**Midfoot**	74.4 ​± ​49.7	16.0–305.8	
**Hindfoot**	173.1 ​± ​128.4	54.3–865.2	
**Contact time, ms**	942.0 ​± ​299.9	158.3–1946.7	67
**Platto-score, median [IQR]**	21.05 [10.5–31.6]	5.26–63.2	69
**ROA location (%)**			75
**Isolated MTP1 OA**	28.0		
**Polyarticular midfoot OA**	40.0		
**Ankle OA**	34.7		
Comorbidity-related factors			
**BMI (kg/m2)**	29.5 ​± ​5.0	20.7–41.3	75
**Comorbidity count, median [IQR]**	4 [[2], [3], [4], [5], [6]]	0–14	75
**Comorbidities (%)**			75
**Cardiorespiratory**	64.0		
**Gastrointestinal**	22.7		
**Urogenital**	36.0		
**Dermatologic**	13.3		
**Neurologic**	45.3		
**General (e.g., endocrine, metabolic, general infections)**	72.0		
**Other**	52.0		
**Anxiety/Depression (HADS), median [IQR]**			75
**Total**	11 [[7], [8], [9], [10], [11], [12], [13], [14], [15], [16], [17], [18], [19], [20]]	0–40	
**Anxiety**	6 [[3], [4], [5], [6], [7], [8], [9], [10], [11]]	0–22	
**Depression**	5 [[2], [3], [4], [5], [6], [7], [8], [9]]	0–20	
**Knee pain (%)**	50.7		75
**Hip pain (%)**	46.7		75

FFI, Foot Function Index.

HADS, Hospital Anxiety and Depression Scale.

PP, Peak Pressure.

PTI, Pressure Time Integral.

ROA, Radiographic Osteoarthritis.

MTP1, 1st metatarsophalangeal joint.

The results of the multivariable regression analyses per sub-category are displayed in Table 2. In the model for the sub-category demographic factors, only female sex was retained (R^2^ ​= ​0.07 [7%], adjusted R^2^ ​= ​0.57 [5.7%]). %). In the sub-category foot-specific factors, five factors were retained: total PP, PP under the midfoot, Platto-score, midfoot pain, and hindfoot pain. The R^2^ was 0.45 (45%) and the adjusted R^2^ was 0.40 (40%). Three factors were retained in the model for the sub-category comorbidity-related factors: BMI, neurologic comorbidity, and HADS score. The R^2^ was 0.426 (42.6%) and the adjusted R^2^ was 0.402 (40.2%).

**Table 2:** Results of multivariable regression analyses per sub-category on the FFI.

	B	95% CI	p
Demographic factors			
**Age**	-.258	-.735 to .218	.283
**Sex, female**	12.926	1.815 to 24.038	**.023**
**Marital status, single**	6.534	−2.860 to 15.929	.170
**Educational attainment**			
**No/Primary education**	11.431	−5.089 to 27.950	.158
**Secondary education**	Reference		
**Higher education**	−1.586	−11.149 to 7.977	.742
**R**^**2**^**= .07 (7%)** **Adjusted R**^**2**^**= .057 (5.7%)**			
Foot-specific factors			
**Forefoot pain**	6.347	−10.015 to 22.709	.440
**Midfoot pain**	11.345	.935 to 21.756	**.033**
**Hindfoot pain**	13.215	4.165 to 22.265	**.005**
**Plantar pressure**			
**PP**			
**Total**	-.015	-.028 to −.001	**.031**
**Forefoot**	.014	-.012 to .039	.287
**Midfoot**	.071	.029 to .113	**.001**
**Hindfoot**	-.017	-.050 to .017	.318
**Contact time**	.001	-.018 to .021	.879
**Platto-score**	55.321	25.665 to 84.977	**<.001**
**ROA location**			
**Isolated MTP1 OA**	1.453	−19.019 to 21.924	.887
**Polyarticular midfoot OA**	2.934	−5.229 to 11.097	.474
**Ankle OA**	.588	−9.860 to 11.036	.911
**R**^**2**^**= 0.451 (45.1%)** **Adjusted R**^**2**^**= 0.403 (40.3%)**			
Comorbidity related factors			
**BMI**	1.756	1.018 to 2.495	**<.001**
**Comorbidity count**	.674	−2.125 to 3.473	.632
**Comorbidities**			
**Cardiorespiratory**	−2.693	−10.895 to 5.510	.514
**Gastrointestinal**	5.981	−2.264 to 14.227	.152
**Urogenital**	3.068	−4.195 to 10.332	.402
**Dermatologic**	−9.704	−20.157 to .750	.068
**Neurologic**	14.603	6.961 to 22.246	**<.001**
**General**	−2.752	−13.756 to 8.252	.619
**Other**	5.754	−1.341 to 12.849	.110
**Anxiety/Depression (HADS)**	.405	.044 to .765	**.028**
**Knee pain**	−2.764	−10.754 to 5.226	.492
**Hip pain**	3.034	−4.655 to 10.723	.434
**R**^**2**^**= .426 (42.6%)** **Adjusted R**^**2**^**= .402 (40.2%)**			

BMI, Body Mass Index.

HADS, Hospital Anxiety and Depression Scale.

PP, Peak Pressure.

ROA, Radiographic Osteoarthritis.

MTP1, 1st metatarsophalangeal joint.

The results of the final bootstrapped multivariable regression analyses are shown in Table 3. Five factors were retained in the final model: female sex, hindfoot pain, higher BMI, neurological comorbidity, and higher HADS score were significantly associated with higher FFI score. The explained variance for the final model (R^2^) was 0.580 (58%), while the adjusted R^2^ was 0.549 (54.9%).

**Table 3:** Bootstrapped results of multivariable regression analysis on the FFI: final model.

	B	95% CI	P
Demographic factors			
**Sex, female**	12.139	3.586 to 20.674	.003
Foot-specific factors			
**Hindfoot pain**	14.628	7.570 to 21.900	.0005
Comorbidity related factors			
**BMI**	1.224	.484 to 2.045	.002
**Neurologic comorbidity**	12.283	5.678 to 18.014	.001
**Anxiety/Depression (HADS)**	.560	.231 to .905	.001
**R**^**2**^**= .580 (58%)** **Adjusted R**^**2**^**= .549 (54.9%)**			

BMI, Body Mass Index.

HADS, Hospital Anxiety and Depression Scale.

## 5 Discussion

The aim of this study was to investigate potential determinants of pain and activity limitations (i.e., demographic, foot-specific, and comorbidity-related factors) in patients with OA of the foot who were referred to a specialized center for rehabilitation and rheumatology. Being female, having pain located in the hindfoot, a higher BMI, neurological comorbidity and a higher HADS score were independently associated with a higher FFI score, indicating a relationship with a higher level of foot-related pain and activity limitations.

Being female was significantly associated with a higher FFI score. This could be the result of 80% of the participants being female. However, the finding of more pain and self-reported activity limitations being more prevalent in women than in men is in accordance with previous research in foot OA [7] and general pain conditions [29]. Of the foot-specific factors, presence of pain located in the hindfoot was associated with a higher FFI score. This could be interpreted as pain located in the hindfoot being more intense and more debilitating than pain in the forefoot. A reason for this could be that the midfoot and hindfoot are exposed to more axial load than the forefoot. It may also be more difficult to avoid loading these parts of the foot, due to their more proximal position.

BMI, neurological comorbidity and HADS score were comorbidity-related factors that were retained in the final model. The finding that higher BMI was associated with worse clinical outcome can be explained through multiple mechanisms: a higher mechanical load on the foot/ankle joints [30], increased levels of low-grade inflammation [31], and a risk for associated comorbidities such as diabetes, cardiovascular diseases, and mental health issues due to overweight/obesity [32,33]. The relationship between BMI and foot health in OA has also been shown in other studies [5,7]. Additionally, in a recent study conducted by Dahmen et al. [34], it was found that a higher BMI was associated with poorer foot health in patients with rheumatoid arthritis. As a high BMI is a modifiable factor, the implications of this finding may be relevant in clinical practice. By focusing on weight loss, pain and activity limitations may be reduced, while also improving other aspects of the individual’s health.

Suffering from neurological comorbidities (or signs/symptoms thereof) was significantly associated with high levels of pain and activity limitations. The neurological conditions prevalent in the present study were stroke/brain aneurysm (6.7%), migraines/severe headaches (25.3%), dizziness (13.3%), and signs/symptoms of neuropathy (reduced sensibility/numbness [37.3%] or feeling of walking on cotton wool [20%]). These diseases and symptoms may lead to increased limitations in daily activities and an intensified experience of pain due to central sensitization or central hypersensitivity [[35], [36], [37]]. Additionally, in neuropathy, pain in the feet is a common symptom. Furthermore, a higher HADS score, indicating more symptoms of anxiety or depression was related to a higher FFI. Psychological distress can have a negative influence on the experience of pain and daily functioning in several conditions [5,[15], [16], [17],^38^].

In the present study, ROA in the foot and/or ankle joints was not associated with pain and activity limitations. This supports the results of other studies that have not found any relationship between radiographic findings and pain in foot OA [10,11,13], and may be an important finding, as radiographs of the foot and ankle play an important role in current diagnostic practices. Also, in OA of other joints it is known that correlations between radiographic findings and clinical symptoms are weak at best [39,40]. It should be noted that there is currently no standardized way of diagnosing foot and/or ankle OA. The current way of diagnosing the condition, in both clinical practice and research, relies on radiograph results. Unfortunately, there are no clinical criteria to adhere to for radiographic results in foot OA, unlike for knee and hip OA. Thus, radiographs in foot OA could result in different decisions on diagnosis, due to the radiograph being taken at different projections or different aspects of the radiograph being assessed (e.g., osteophytes or joint space narrowing). In the context of research, this leads to problems with comparability of studies and generalizability of the results. It may, however, be added that a standardized approach to radiographically assess OA of five joints in the forefoot and midfoot in a research context has been suggested by Menz et al. [13].

Plantar pressure variables were not found to be independently associated with pain and activity limitations in the present study, even though higher midfoot plantar pressure was found to be related in the first step of the analysis. Previous studies in foot OA have shown conflicting evidence with regards to this [14,41]. However, a relationship between pain and increased plantar pressure has been reported in literature on rheumatoid arthritis of the foot [e.g., 42]. Although the two conditions differ in many ways, it is plausible that such a correlation may be present also in foot OA. It has, however, also been suggested that lower plantar pressure may be related to pain, resulting from an attempt to avoid additional load on painful joints [43]. Further studies are needed to determine the relationship between plantar pressure and pain and activity limitations. As plantar pressure is modifiable, it may be an important factor in the treatment of foot symptoms.

A strength of the present study is that a large variety of factors have been investigated in relation to the FFI score. As an exploratory study, the relationships found in the present study lay a foundation for further research on determinants of foot-related pain and activity limitations in OA. Some limitations should also be considered when interpreting the results. Firstly, the sample was rather small relative to the number of variables in the model, we therefore used regression analyses in two steps. Secondly, as the sample was recruited from a specialized rehabilitation center, it is likely that these patients had more complex and advanced complaints than patients in primary care. To be able to generalize across these groups, future studies should include a broader spectrum of patients from different care levels. Thirdly, in the current study, the patients were referred from the clinic and were admitted based on the clinical diagnosis, and therefore, X-ray views and X-ray reports were not taken in a protocolized way. Most X-rays were taken dorso-lateral, lateral and weight-bearing. This may have affected the diagnosis given, and thus the inclusion in the study. Fourthly, although many potential determinants were included in this study, there are still many others that could play a significant role in the perception of pain and activity limitations in this patient group. Level of physical activity and type of shoes are examples of factors that should be included in future studies on this topic. Finally, cross-sectional data were used, and as such, no conclusions on causality can be drawn.

## 6 Conclusion

Female sex, pain located in the hindfoot, higher BMI, neurological comorbidity, and greater psychological distress are independently associated with a higher level of foot-related pain and activity limitations. By addressing these determinants in the management of foot OA, pain and activity limitations can potentially be reduced. Further observational and interventional research is needed on this subject.

## Author contributions

VFM Ryman (FR), M van der Leeden (MvdL), M van der Esch (MvdE), and LD Roorda (LDR) took part in the conception and design of the study, analysis and interpretation of the data, and the final approval of the article. SK Verberne (SKV) took part in the data collection. FR created the first draft of the article, and MvdL, MvdE,LDR, and SKV contributed with critical revision for important intellectual content.

J Dekker (JD), J van Dieën (JvD), JWR Twisk (JT), E Huijbrechts (EH), and WF Lems (WFL) contributed with interpretation of the data, critical revision of the article for important intellectual content, and the final approval of the article.

## Declaration of funding and role of the funding source

No funding was provided for the present study.

## Declaration of competing interest

The authors have no competing interests to declare.
